# Validation of the Prolonged Grief Disorder-13-Revised in a Sample of Older Adults

**DOI:** 10.1093/geroni/igaf122.2875

**Published:** 2025-12-31

**Authors:** Hannah Apostolou, Lindsey Jacobs, Rebecca Allen, A Lynn Snow, Alexa Tullett, Hyunjin Noh

**Affiliations:** The University of Alabama, Milwaukee, Wisconsin, United States; The University of Alabama, Tuscaloosa, Alabama, United States; The University of Alabama, Tuscaloosa, Alabama, United States; The University of Alabama, Tuscaloosa, Alabama, United States; The University of Alabama, Tuscaloosa, Alabama, United States; The University of Alabama, Tuscaloosa, Alabama, United States

## Abstract

Older adults are at an increased risk of developing Prolonged Grief Disorder (PGD) due to the heightened chances of experiencing a loss, lower levels of cognitive functioning, and heightened rates of loneliness and depression. Currently, the Prolonged Grief Disorder-13 Revised (PG-13-R) is the only measure specifically designed to assess for PGD. The PG-13-R was developed to reduce misdiagnoses of PGD and avoid pathologizing normal grief, as research has found grief to be a distinct psychological response to bereavement. The present study compared the PG-13-R to other measures of similarly presenting mental health disorders. Our sample consisted of 120 racially and ethnically diverse adults aged 50 and older who completed measures of grief, depression, anxiety, post-traumatic stress disorder (PTSD) and loneliness using REDCap. Correlation coefficients were computed using SPSS Version 29 to investigate convergent and discriminant validity of the PG-13-R and other mental health disorder measures. The results showed the PG-13-R was strongly correlated with the PTSD measure (r = 0.67, p < 0.001), moderately correlated with the anxiety measure (r = 0.49, p < 0.001), and weakly correlated with measures of depression (r = 0.39, p < 0.001) and loneliness (r = 0.34, p < 0.001). This suggests the PG-13-R and PTSD measure are capturing aspects of a related concept. The PG-13-R could be further refined to capture unique features of grief, distinct from PTSD, to improve the quality of grief assessment. Continuing to improve existing measures further equips mental health professionals to serve the community by advancing the identification, assessment, and treatment of mental health disorders.

